# Characteristics and Resistance to Cisplatin of Human Neuroblastoma Cells Co-Cultivated with Immune and Stromal Cells

**DOI:** 10.3390/bioengineering9110655

**Published:** 2022-11-05

**Authors:** Kristina V. Kitaeva, Daria S. Chulpanova, Margarita N. Zhuravleva, Ivan Yu. Filin, Ruslan M. Deviatiiarov, Alyssa C. Ballard-Reisch, Albert A. Rizvanov, Valeriya V. Solovyeva

**Affiliations:** Institute of Fundamental Medicine and Biology, Kazan Federal University, 420008 Kazan, Russia

**Keywords:** co-culture, mesenchymal stromal cells, mononuclear cells, neuroblastoma, tumor microenvironment, extracellular matrix, test system, cisplatin, cytokine profile

## Abstract

We investigated the features of the morphology and cytokine profiles of neuroblastoma SH-SY5Y cells, bone marrow-derived mesenchymal stromal/stem cells (BM-MSCs), and peripheral blood mononuclear cells (PBMCs) in double (BM-MSCs + SH-SY5Y cells) and triple (BM-MSCs + SH-SY5Y cells + PBMCs) co-cultures incubated on plastic and Matrigel. Cells in the co-cultures communicated by vesicular transport and by exchanging membrane and cytoplasmic components. The cytokine profile of double and triple co-cultures incubated on Matrigel and plastic had differences and showed the highest concentration of a number of chemokines/cytokines, such as CXCL8/IL-8, I-TAC/CXCL11, IP10/CXCL10, MDC/CCL22, MIP-1α/CCL3, IL-1β, ENA-78/CXCL5, Gro-α/CXCL1, MCP-1/CCL2, TERC/CCL25, CXCL8/IL-8, and IL-6. High concentrations of inflammatory chemokines/cytokines in the conditioned medium of triple co-culture form a chronic inflammation, which brings the presented co-cultivation system closer to a natural tumor. Triple co-cultures were more resistant to cisplatin (CDDP) than the double- and monoculture of SH-SY5Y. The mRNA levels of *BCL2, BCL2L1, RAC1, CAV1, CASP3,* and *BAX* genes were changed in cells after co-culturing and CDDP treatment in double and triple co-cultures. The expression of the BCL2, BAX, CAV1, and CASP3 proteins in SH-SY5Y cells after the triple co-culture and CAV1 and BAX protein expression in SH-SY5Y cells after the double co-culture were determined. This study demonstrated the nature of the cellular interactions between components of tumor niche and the intercellular influence on chemoresistance observed in our tumor model, which should enable the development of novel test systems for anti-tumor agents.

## 1. Introduction

Neuroblastoma is one of the most common extracranial solid tumors, usually occurring in children under 2 years of age, and 90% of cancer patients in this group are under 5 years old [1]. Approximately 50% of patients have metastases in the bones, bone marrow, lymph nodes, and other soft tissues [2]. The tumor stroma plays a key role in tumor development and progression, which also have a profound effect on anti-cancer drug efficacy [3]. Within the tumor microenvironment (TME), cells acquire abnormal phenotypes and functions mediated by complex cell–cell interactions, including direct cell–cell, paracrine factors, and other biologically active molecules such as microvesicles (MVs) and their cargos (nucleic acids and proteins) [4]. The composition of the tumor stroma usually consists of immune cells (macrophages, lymphocytes, NK cells, and neutrophils), endothelial cells, myofibroblasts and cancer-associated fibroblasts, adipocytes, and an extracellular matrix (ECM) [5]. A large body of research has focused on cancer-associated macrophages (CAFs) due to their importance in supporting the growth and vascularization of tumors, inducing epithelial–mesenchymal transition (EMT), and enhancing tumor cell invasion. Studies conducted using xenograft glioma mouse models have shown a high degree of tropism of mesenchymal stromal/stem cells (MSCs) toward tumor regions [6]. The tumor stroma appears as a type of chronic inflammation, facilitating the engagement and recruitment of tumor stem cells [6,7]. Upon entry into the tumor stroma, MSCs can differentiate into more mature mesenchymal cells, such as CAFs, macrophages, or endothelial cells [8], the functions of which consist of the creation of a supportive microenvironment for tumor growth.

Moreover, tumors can drive the generation of immunosuppressive or regulatory immune cells, as well as recruit a large number of tumor-promoting myeloid cells to establish the TME, thus promoting tumor progression. Compelling evidence indicates that myeloid-derived suppressor cells, regulatory T cells (Treg), tumor-associated macrophages, regulatory dendritic cells, neutrophils, T-helper 17 cells (Th17), and regulatory B-cells are key immunosuppressive cells that promote tumor progression [9]. For example, macrophages, differentiated from monocytes, are involved in the immune response to the infection and demonstrate anti-tumor functionality under normal circumstances (M1 phenotype). However, in hypoxic, inflammatory, and otherwise abnormal TMEs, macrophages can adopt the tumor-promoting phenotype M2, which promotes cancer cell proliferation, immunosuppression, and angiogenesis in support of tumor growth and metastasis [10]. The common type of white cells in circulation (neutrophils) can also adopt both an anti-tumor (N1) and a pro-tumor (N2) phenotype in tumor response [11].

Current screening methods for antitumor drugs, such as NCI60, do not take into account the complex interactions of tumor cells with stromal and immune microenvironment cells and the ECM [12]. Thus, to reproduce the processes occurring in the tumor stroma, realistic in vitro tumor models which recreate the co-cultures of different cell types found within the TME need to be developed. The development of such models is not only essential to advance our understanding of the complex role played by the microenvironment in cancer progression but is also required for the evaluation of drug efficacy for future therapeutic use. The processes of metastases formation and tumor progression in the stromal microenvironment are growing areas of interest, specifically the selection of effective antitumor drugs for the treatment of oncological diseases.

We characterized the cell interactions in double co-culture consisting of MSCs and neuroblastoma SH-SY5Y cells and triple co-culture consisting of MSCs, peripheral blood mononuclear cells (PBMCs), and SH-SY5Y cells and the changes in the secreted cytokines of co-cultured cells. Moreover, we evaluated the effect of co-culturing on proliferative activity and *BAX, BCL2, BCL-XL, CAV1, CASP3,* and *RAC1* gene expression after cisplatin (cis-diamminedichloroplatinum-II, CDDP) treatment.

## 2. Materials and Methods

### 2.1. Cell Cultures

For MSC isolation, the human femoral head (provided by the Republican Clinical Hospital for research purposes in compliance with ethical standards and in accordance with the current legislation; the protocol was approved by the Biomedicine Ethic Expert Committee of Kazan Federal University (#3, 23.03.2017)) was crushed for bone marrow extraction using sterile instruments in a laminar box and mixed with the nutrient medium DMEM/F12 (PanEco, Moscow, Russia) containing 10% fetal bovine serum (FBS) (HyClone, Logan, UT, USA), 2 mM of L-glutamine (PanEco, Moscow, Russia), and a mixture of antibiotics penicillin (100 U/mL) and streptomycin (100 μg/mL) (PanEco, Moscow, Russia) (complete DMEM/F12 medium). The resulting mixture was poured into a sterile adhesive T-75 flask and incubated at 37 °C in a humidified atmosphere with a content of 5% CO_2_. After 48 h of cultivation, non-adherent cells were removed; the culture medium was replaced twice a week. MSCs isolated from bone marrow (BM-MSCs) were harvested after reaching ≥80% confluence using trypsin-EDTA solution (PanEco, Moscow, Russia).

PBMCs were isolated from the whole blood of a healthy volunteer donor (provided by the Republican Clinical Hospital for research purposes in compliance with ethical standards and in accordance with the current legislation; the protocol was approved by the Biomedicine Ethic Expert Committee of Kazan Federal University (#3, 23.03.2017)). Peripheral blood (5 mL) was mixed under aseptic conditions with DPBS solution (PanEco, Moscow, Russia) in a 1:2 ratio. Ficoll solution with a density of 1.077 g/cm^3^ (PanEco, Moscow, Russia) was poured into another clean tube filled with a similar volume. The mixture of blood and DPBS was neatly layered on the Ficoll solution, avoiding mixing, and then centrifuged at 1900 rpm for 20 min. After centrifuging, the mononuclear fraction was carefully pipetted into a clean conical tube and washed with DPBS solution at 1400×*g* rpm for 5 min. After the cells were resuspended in IMDM nutrient medium (PanEco, Moscow, Russia) (containing 10% FBS, 2 mM of L-glutamine, and a mixture of penicillin (100 U/mL) and streptomycin (100 µg/mL)) at 37 °C in a humidified atmosphere with a content of 5% CO_2_.

Human neuroblastoma SH-SY5Y cells were purchased from the American Type Culture Collection (ATCC number: CRL-2266, Manassas, VA, USA). Co-cultures were established by plating 1:1 for double co-culture or 1:1:1 for triple co-culture ratios of BM-MSCs and SH-SY5Y cells or BM-MSCs, PBMCs, and SH-SY5Y cells. Co-cultures were maintained in DMEM with 10% FBS at 37 °C in a humidified atmosphere of 5% CO_2_.

### 2.2. Immunophenotyping and Differentiation of BM-MSCs

Immunophenotyping of BM-MSCs was performed using the BD Stemflow™ Human MSC Analysis Kit (BD Biosciences, San Jose, CA, USA). Additionally, antibodies to CD29 (#303007, BioLegend, San Diego, CA, USA) and CD166 (#2319515, Sony Biotechnology, San Diego, CA, USA) were also used. Isotype-matched nonreactive fluorochrome-conjugated antibodies were used as controls. The cell populations were analyzed using FACS Aria III (BD Biosciences, San Jose, CA, USA), as previously described [13], and the data were analyzed using BD FACSDiva™ software version 7.0. Differentiation was performed using a StemPro^®^ Adipogenesis Differentiation Kit (#A10070-01, Gibco, Grand Island, NY, USA), StemPro^®^ Osteogenesis Differentiation Kit (#A10072-01, Gibco, Grand Island, NY, USA), and StemPro^®^ Chondrogenesis Differentiation Kit (#A10071-01, Gibco, Grand Island, NY, USA) for adipogenic, osteogenic, and chondrogenic differentiation, according to the manufacturer’s instructions. On the 18th day of differentiation, cells were stained by different protocols described below. Cells differentiated in the adipogenic direction were stained for neutral lipids using Oil Red O (#O0625, Sigma-Aldrich, St. Louis, MO, USA) for 30 min and Richard-Allan Scientific™ Modified Mayer’s Hematoxylin (#72804, Epredia, Kalamazoo, MI, USA) for 1 min. Cells differentiated in the osteogenic direction were stained according to the von Kossa protocol using 2% silver nitrate solution (AgNO_3_) (#209139, Sigma-Aldrich, St. Louis, MO, USA) for 10 min and incubated under a bright light source for 15 min. After differentiation in the chondrogenic direction, the cells were stained to visualize the acid mucopolysaccharides using an Alcian Blue solution (#A5268, Sigma-Aldrich, St. Louis, MO, USA) for 1 h. Stained cells were analyzed on an inverted microscope Axio Observer.Z1 (Carl Zeiss, GmbH, Germany) with the use of AxioVision v.4.8. software.

### 2.3. Obtaining of GFP-Modified SH-SY5Y Cells

Three types of plasmids were used to create recombinant viruses: vector plasmid pLenti CMV GFP Blast (#17445, Addgene, Watertown, MA, USA) encoding the green fluorescent protein (*gfp*) gene, packaging plasmid psPAX2 (#12260, Addgene, Watertown, MA, USA) and envelope plasmid pMD2-VSV-G (#12259, Addgene, Watertown, MA, USA). A packaging cell line HEK293FT was used, cultivated on 10 cm Petri dishes in the complete DMEM/F12 medium. After lentiviral transfection, the concentration of lentiviral particles was performed using an Optima L-90K Ultracentrifuge (Beckman Coulter, San Diego, CA, USA) at 26,000 rpm and 4 °C for 2 h. Native SH-SY5Y cells were seeded on 6-well plates. The mix of 1 mL of the virus and 3 μL of protamine sulfate solution (Sigma, St. Louis, MO, USA) (at a concentration of 0.25 μg/mL) was added to the cells on 6-well plates, and the medium with viruses was replaced with the complete DMEM/F12 medium after 24 h. After 48 h, the efficacy of lentiviral transduction was evaluated using a fluorescence microscope. Fluorescence indicated the expression of the *gfp* reporter gene and the transduction of cells. The selection of genetically modified cells was performed using flow cytometry. Cells were detached from the culture plastic using the trypsin-EDTA solution, washed from the medium, and resuspended in DPBS. Cells were sorted by *gfp* fluorescence using FACS Aria III (BD Biosciences, San Jose, CA, USA) according to the fluorescence spectrum.

### 2.4. Cell Labeling

BM-MSCs, SH-SY5Y cells, and PBMCs were labeled according to fluorescence using Vybrant DiD (red spectrum), DiI (yellow spectrum), and DiO (green spectrum) for triple co-culture, and SH-SY5Y cells were labeled with DiO (green spectrum) for double co-culture. Trypsinized cells were (1400 rpm for 5 min) and resuspended in serum-free medium prior to quantification. The labeling of cell membranes was conducted using the Vybrant™ Multicolor Cell-Labeling Kit according to the manufacturer’s instructions (#22889, Invitrogen, Waltham, MA, USA) and as previously described [14]; the labeling was verified by fluorescent microscopy using an Axio Observer.Z1 inverted microscope (Carl Zeiss, GmbH, Germany). SH-SY5Y cells were transduced with the lentivirus encoding *gfp* for confocal microscopy assay. BM-MSCs (DiD) and SH-SY5-GFP were seeded on cultural glasses, after co-cultivation cells were fixed with 10% buffered formalin (#06-001/M, BioVitrum, St. Petersburg, Russia) and stained by DAPI (#62247, Invitrogen, Waltham, MA, USA) for nuclear visualization.

### 2.5. Preparation of Matrigel-Coated Plates

After thawing on ice, 200 μL of Matrigel Basement Membrane Matrix (BD Biosciences, San Jose, CA, USA) was placed into each well of a 12-well plate on ice using cooled pipette tips. For Matrigel polymerization, plates were incubated at 37 °C for 30 min before cell seeding.

### 2.6. Fluorescence and Confocal Microscopy

The cell morphology and self-organization of double co-culture consisting of BM-MSCs and SH-SY5Y and triple co-culture consisting of SH-SY5Y cells, BM-MSCs, and PBMCs were analyzed with an inverted fluorescent microscope Axio Observer.Z1 and AxioVision Rel.4.8 software (Carl Zeiss, GmbH, Germany). The cell morphology and intercellular interactions were analyzed with an inverted microscope LSM 780 and Zen black 2012 software (Carl Zeiss, GmbH, Germany).

### 2.7. Fluorescence-Activated Cell Sorting (FACS) of Cells from Double and Triple Co-Cultures

After 96 h of co-culture, cells were trypsinized (Trypsin-EDTA solution, PanEco, Moscow, Russia) and washed with the DPBS to remove residual extracellular matrix. Cells were pelleted between wash steps (1400× *g* rpm for 5 min). Subsequently, cells were sorted according to the fluorescence spectrum and size, using a FACSAria III flow cytometer (BD Biosciences, San Jose, CA, USA).

### 2.8. Cytokine Profile

After 24, 48, and 72 h of cultivation, conditioned medium (CM) from the cells was collected into 1.5 mL conical tubes and centrifuged at maximum speed. The supernatant was taken into separate tubes and 100 μL aliquots were made. Samples were stored at −80 °C. Cytokine levels were analyzed with Bio-Plex Pro Human Chemokine 40-plex Panel (#171AK99MR2, Bio-Rad, Hercules, CA, USA) in CM and co-cultivated on plastic and Matrigel cells for 24, 48, and 72 h. The following analytes were analyzed: 6Ckine/chemokine (C-C motif) ligand (CCL) 21, B cell-attracting chemokine 1 (BCA-1)/chemokine (C-X-C motif) ligand (CXCL) 13, cutaneous T cell-attracting chemokine (CTACK)/CCL27, epithelial neutrophil-activating protein 78 (ENA-78)/CXCL5, eotaxin/CCL11, eotaxin-2/CCL24, eotaxin-3/CCL26, fractalkine/chemokine (C-X3-C motif) ligand (CX3CL) 1, granulocyte chemotactic protein 2 (GCP-2)/CXCL6, GM-CSF, Gro-α/CXCL1, Gro-β/CXCL2, I-309/CCL1, IFN-γ, IL1β, IL2, IL4, IL6, IL8/CXCL8, IL10, IL16, IFN-γ-induced protein 10 (IP-10)/CXCL10, IFN-inducible T cell alpha chemoattractant (I-TAC)/CXCL11, monocyte chemoattractant protein (MCP)-1/CCL2, MCP-2/CCL8, MCP-3/CCL7, MCP-4/CCL13, macrophage-derived chemokine (MDC)/CCL22, macrophage migration inhibitory factor (MIF), monokine induced by IFN-γ (MIG)/CXCL9, MIP-1α/CCL3, MIP-1δ/CCL15, MIP-3α/CCL20, MIP-3β/CCL19, myeloid progenitor inhibitory factor (MPIF)-1/CCL23, small-inducible cytokine B16 (SCYB16)/CXCL16, SDF-1α+β/CXCL12, thymus activation regulated chemokine (TARC)/CCL17, and thymus-expressed chemokine (TECK)/CCL25, TNF-α. A total of 50 μL of the sample of CM was used for determining cytokine concentration and the collected data were analyzed using a Luminex 200 analyzer with MasterPlex CT control software and MasterPlex QT analysis software (MiraiBio division of Hitachi Software, San Francisco, CA, USA).

### 2.9. Cell Proliferation Assay

To analyze the proliferative activity of cells in double (BM-MSCs + SH-SY5Y) and triple (BM-MSCs + SH-SY5Y + PBMCs) co-cultures, the cells were seeded with a density of 1500 cells/well on the 96-well Matrigel-coated plates and uncovered cultural 96-well plates in DMEM supplemented with 10% FBS. The cells were mixed 1:1 for double and 1:1:1 for triple co-culture with the same density. After 48 hours of incubation, the media was replaced and CDDP was added at a concentration of 10 µg/mL (n = 5) (Cisplatin-LANS, LANS-Pharm Ltd., Moscow, Russia). After 72 hours of incubation with CDDP, an MTS test was performed using a CellTiter 96^®^ AQueous Non-Radioactive Cell Proliferation Assay Kit (Promega, San Luis Obispo, CA, USA) to determine the proliferative activity of cells according to the manufacturer’s recommendations.

### 2.10. Quantitative Polymerase Chain Reaction (qPCR)

Total RNA was extracted from BM-MSCs, PBMCs, and SH-SY5Y cells using TRIzol Reagent (Invitrogen, Carlsbad, CA, USA) following the manufacturer’s instructions. Primers and probes specific to 18S ribosomal RNA (18S rRNA), B-cell Lymphoma protein-2 (BCL-2), Bcl-2-like protein 1 (BCL2L1), RAC1, caveolin 1 (CAV1), caspase-3 (CASP3), BCL2 associated X, and apoptosis regulator (BAX) cDNAs were designed by GenScript Online Real-time PCR (TaqMan) Primer Design Tool (GenScript, Piscataway, NJ, USA) and synthesized by Lytech (Moscow, Russia) (Table 1). The expression levels of *BCL2, BCL2L1, RAC1, CAV1, CASP3,* and *BAX* genes were determined by TaqMan quantitative PCR with the following components: 1 μL cDNA template, 0.3 μL of primers and probe mix (final primer concentration of 300 nM each), 6.7 μL of sterile H_2_O (Evrogen, Moscow, Russia), and 2 μL of 5× qPCRmix-HS buffer (Evrogen, Moscow, Russia), at a final volume of 10 μL. The reaction was carried out using the CFX96 Touch™ Real-Time PCR Detection System (BioRad, Hercules, USA) with the following protocol: pre-denaturation at 95 °C for 1 min, 44 cycles of denaturation at 95 °C for 30 s, annealing at 55 °C for 30 s, and extension at 72 °C for 10 min. The relative expression levels of target genes were calculated by the ΔΔCT method with 18S rRNA as a reference gene (Table 1).

### 2.11. Western Blot Analysis

Total protein was extracted from BM-MSCs, PBMCs, and SH-SY5Y cells using RIPA Lysis Buffer (Thermo Fisher Scientific, San Jose, CA, USA) and a centrifugation series. Then, 15 μg of total protein was prepared for each sample, separated in 4–12% acrylamide gels, and transferred onto PVDF membranes (Bio-rad, San Jose, CA, USA). PVDF membranes were incubated in 5% milk–TBST (Tris Buffer Solution, 0.1% Tween-20, Sigma-Aldrich, St. Louis, MO, USA), incubated with primary antibodies in 5% milk–TBST, and then washed in TBST. After incubation with primary antibodies, the blots were incubated with appropriate HRP-conjugated secondary antibodies (#ab205719, Abcam, Fremont, CA, USA). Chemiluminescent detection was performed using Pierce™ ECL Western Blotting Substrate (Bio-Rad, San Jose, CA, USA). Chemiluminescence was detected with a ChemiDoc XRS^+^ system; images were acquired with the ImageLab software v6.1 (Bio-Rad, San Jose, CA, USA). The primary antibodies used in this study were: anti-Caspase-3 antibody (#ab4051, Abcam, Fremont, CA, USA), anti-Bcl2 antibody (#ab59348, Abcam, Fremont, CA, USA), Anti-BAX antibody (#ab104156, Abcam, Fremont, CA, USA), Anti-Caveolin-1 antibody (#ab2910, Abcam, Fremont, CA, USA), and THE™ beta Actin Antibody (HRP) (#A00730, GenScript, Redmond, WA, USA). Dilution was performed according to manufacturer instructions.

### 2.12. Statistical Analysis

Using GraphPad Prism 7 software (GraphPad Software), the MTS assay and qPCR data were analyzed through one-way ANOVAs, followed by Tukey HSD post hoc comparison tests. Significant probability values are denoted as * *p* < 0.05, ** *p* < 0.01, *** *p* < 0.001, and **** *p* < 0.0001, and the standard deviation (SD) is shown. The statistical analysis and visualization of cytokine profile data were carried out in the R development environment. A logarithm (log2) of the average value in the experiment was used to construct a distance matrix and cluster and visualize a heat map. Stats v3.5.1 (dist and hclust functions) and the gplots v3.0.1.1 (heatmap.2 function) packages for R were used.

## 3. Results

### 3.1. BM-MSCs, SH-SY5Y Cells, and PBMCs Interact in Double and Triple Co-Cultures, Vesicular Transport, and Exchange of the Membrane and Cytoplasmic Components

**Figure 1:**
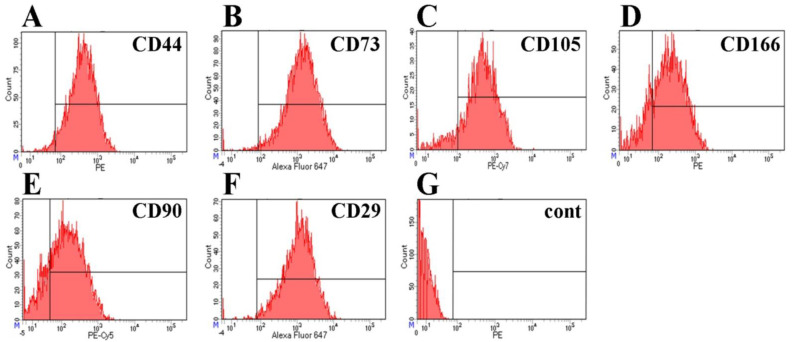
Immunocytofluorimetric characterization of surface antigens (CD markers) of BM-MSCs. (**A**) CD44 (95.9% of positive cells), (**B**) CD73 (98.2%), (**C**) CD105 (88.6%), (**D**) CD166 (79.6%), (**E**) CD90 (79.2%), (**F**) CD29 (98.5%), (**G**) negative control: CD34, CD11b, CD19, CD45, and HLA-DR (0% positive cells).

**Figure 2:**
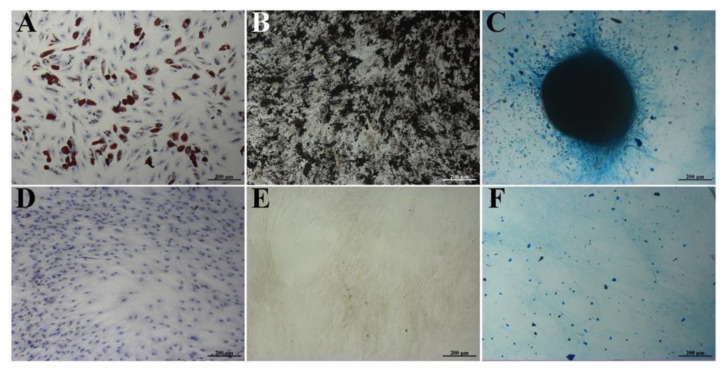
Differentiation of isolated BM-MSCs into adipogenic (**A**), osteogenic (**B**), and chondrogenic (**C**) lineages, as verified by Oil Red O, von Kossa, and Alcian Blue staining, respectively. (**A**–**C**) Differentiation media. (**D**–**F**) Complete DMEM media. Scale bar: 200 µm.

The immunophenotype of isolated BM-MSCs showed no markers of differentiated immune cells in the population (CD34, CD11b, CD19, CD45, HLA-DR), but showed the presence of antigens typical for MSCs—CD44, CD73, CD166, CD90, CD29, and CD105 (Figure 1)—confirming that the MSC cultures were free of other differentiated cell types. As a result of differentiation in the adipogenic direction, a large number of neutral lipid droplets in the cells was observed after staining (identified by Oil Red O staining (Figure 2A)). After differentiation in the osteogenic direction, inclusions of insoluble calcium phosphate were detected in cells. Additionally, changes in the shape of the cells from the fusiform to the cuboidal and the polygonal characteristic of osteocytes were also observed. BM-MSCs cultured with the control medium demonstrated no changes in the morphology of the cells or their content (Figure 2B). After differentiation in the chondrogenic direction, the formation of cartilage structures positively stained with acidic mucopolysaccharides, characteristic of cartilage tissue, was observed in the differentiated cells in contrast to the control cells (Figure 2C).

**Figure A1:**
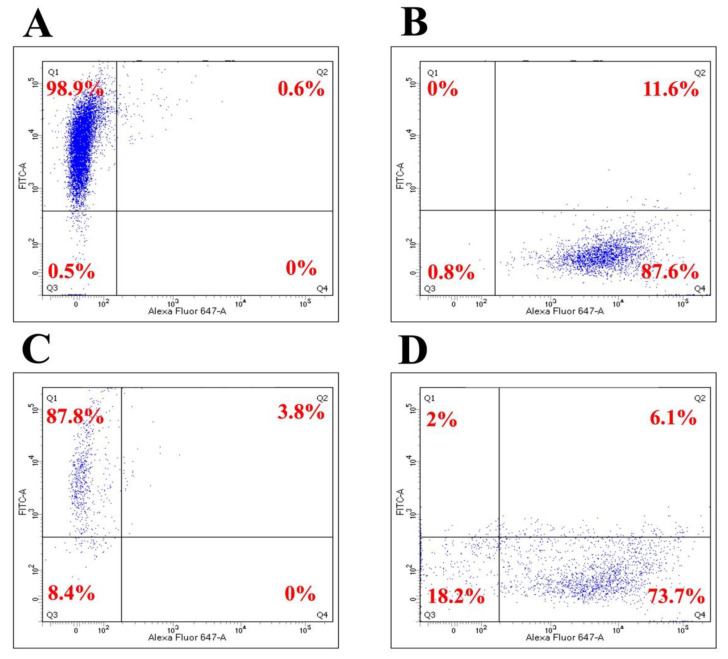
Flow cytometric analysis of SH-SY5Y cells and BM-MSCs colored with vital dye. SH-SY5Y cells colored with DiO dye (**A**,**C**), BM-MSCs (monoculture) colored with the vital DiD dye after cultivation for 96 h. (**A**,**B**) Plastic, (**C**,**D**) Matrigel. Q1—population of cells with green fluorescence (DiO). Q2—population of cells with mixed fluorescence (DiO + DiD). Q3—unstained population. Q4—population of cells with red fluorescence (DiD).

The incubation of BM-MSCs and SH-SY5Y cells with Vybrant DiD (red) and Vybrant DiO (green) fluorescent dyes, respectively, in double co-culture, and the incubation of BM-MSCs, SH-SY5Y cells, and PBMCs with Vybrant DiD (red), Vybrant DiI (yellow), and Vybrant DiO (green) fluorescent dyes in triple co-culture, respectively, resulted in the labeling of the majority of the cells (data shown in Appendix A, Figure A1), enabling the different cell types to be distinguished in the co-culture.

**Figure 3:**
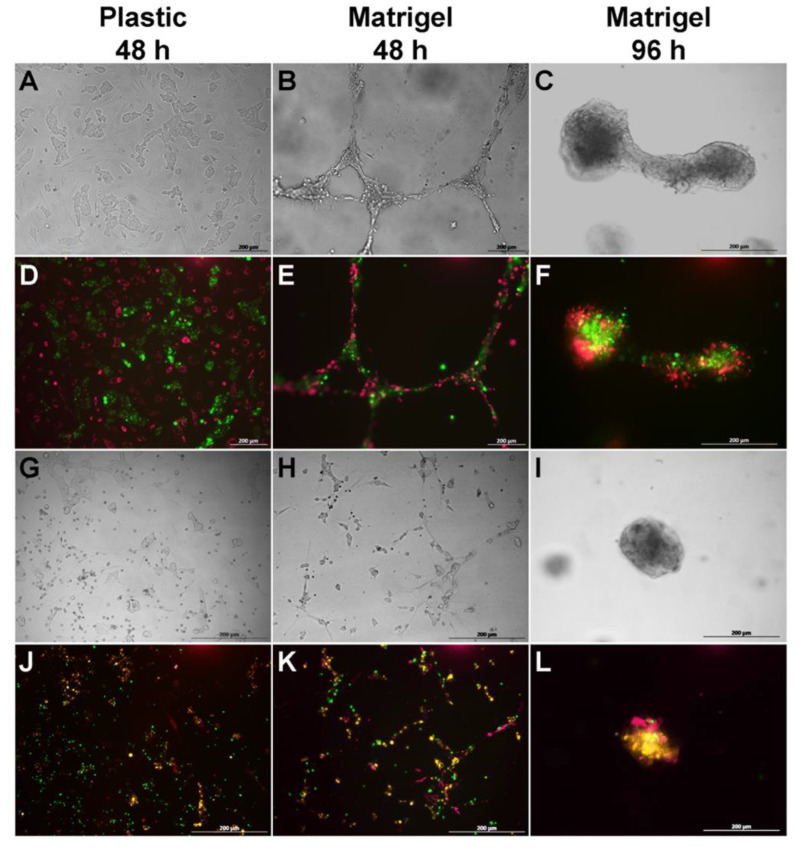
Self-organization of BM-MSCs, PBMCs, and SH-SY5Y cells in co-culture on untreated plastic and Matrigel. (**A**–**C**,**G**–**I**) Phase contrast light microscopy. (**D**–**F**,**J**–**L**) Fluorescence microscopy. (**A**–**F**) Double co-culture contains SH-SY5Y cells (FITC, green) and BM-MSCs (Alexa Fluor 647, red). (**G**–**L**) Triple co-culture contains SH-SY5Y cells (PE, yellow), BM-MSCs (Alexa Fluor 647, red), and PBMCs (FITC, green). (**D**–**F**,**J**–**L**) Fluorescence microscopy: alignment of the fluorescence spectra of Alexa Fluor 647 (BM-MSCs, red), PE (SH-SY5Y cells, yellow), and FITC (PBMCs, green). Scale: 200 µm.

**Figure 4:**
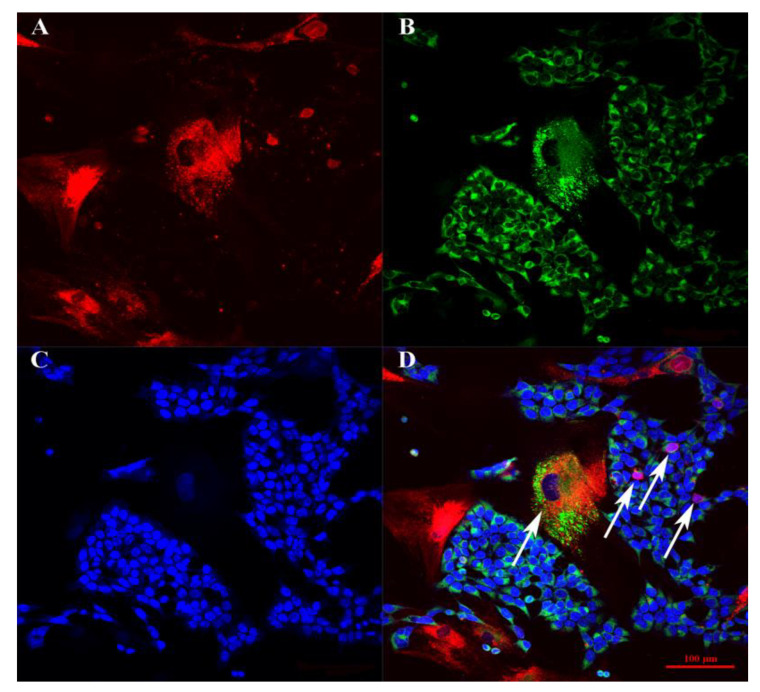
The confocal microscopy BM-MSCs and SH-SY5Y cells on glasses. (**A**) Channel filtered for the emission spectrum of Alexa Fluor 647. (**B**) Channel filtered to gather fluorescence emission from Alexa Fluor 488. (**C**) Channel filtered for the emission spectrum of Alexa Fluor 460. (**D**) Merge. Arrows indicate the cells that have mixed colors as a result of membrane and cytoplasmic exchange between cells.

When BM-MSCs and SH-SY5Y cells were co-cultured for 48–96 h on plastic plates, SH-SY5Y cells formed islets in the BM-MSC monolayer, which formed canal-like structures (Figure 3B,E). In the triple co-culture, a similar self-organization of BM-MSCs and SH-SY5Y cells was observed, as well as the attraction of PBMCs around the islets of SH-SY5Y cells (Figure 3). The incubation of BM-MSCs and SH-SY5Y cells for 48 h on Matrigel resulted in the formation of capillary-like structures consisting of a mixed culture of tumor cells and BM-MSCs (Figure 3). Similar capillary-like structures were observed in the triple culture, SH-SY5Y cells, BM-MSCs, and PBMCs (Figure 3H,K). However, after 96 h of incubation, the cells of both double and triple co-cultures changed their self-organization and formed spheroids (Figure 3F,L). The confocal microscopy assay revealed a high exchange of membrane and cytoplasmic components between BM-MSCs and SH-SY5Y (Figure 4).

**Figure 5:**
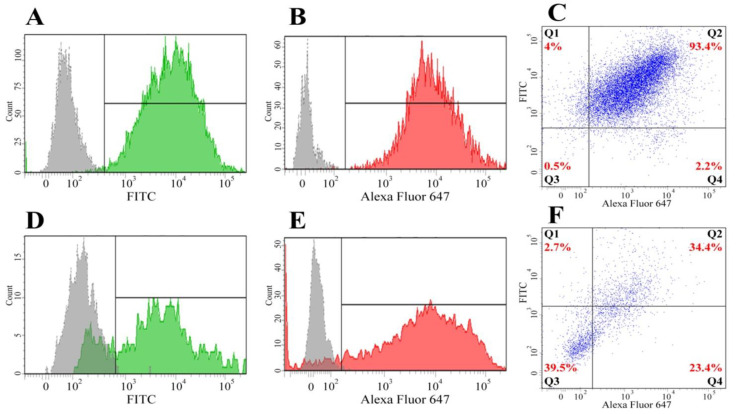
Flow cytometry analysis of BM-MSCs and SH-SY5Y cells after co-culture for 96 h. (**A–C**) Plastic. (**D–F**) Matrigel. (**A**,**D**) SH-SY5Y cells (monoculture) colored with vital DiO dye. (**B**,**E**) BM-MSCs (monoculture) colored with the vital DiD dye. (**C**,**F**) BM-MSCs and SH-SY5Y cells after co-cultivation (co-culture). Q1—population of cells with green fluorescence (DiO). Q2—population of cells with mixed fluorescence (DiO + DiD). Q3—unstained population. Q4—population of cells with red fluorescence (DiD).

After 96 h of BM-MSC and SH-SY5Y co-cultivation, flow cytometry analysis of the selected cell populations showed a significant exchange of membrane components between the cell cultures. For example, by culturing BM-MSCs and SH-SY5Y cells on plastic, 93.4% of the cells had two fluorescence spectra (DiO + DiD, FITC + AlexaFluor-645) (Figure 5). The population of cells within the green fluorescence spectrum was 4%, red was 2.2%, and the unstained population was 0.5% (Figure 5C).

When BM-MSCs and SH-SY5Y cells were cultured for 96 h on Matrigel, the flow cytometry analysis showed the presence of cells with mixed fluorescence (34.4%). The population of the cells within the green fluorescence spectrum was 2.7%, red was 23.4%, and the unstained population was 39.5% (Figure 5). In comparison with the co-culture on plastic, the joint culture on Matrigel showed a greater number of unstained cells (0.5% vs. 39.5%). Since initial cultures were mostly stained (>93%), the appearance of cells with staining below the detection threshold can probably be explained by a “dilution” effect due to the loss of the label through proliferation or by the label transfer to the ECM. The size and granularity of the cell populations after co-cultivation on plastic and Matrigel showed no significant change (Figure 5C,F).

### 3.2. Co-Cultivation Leads to Changes in the Cytokine Profile of BM-MSCs, SH-SY5Y Cells, and PBMCs

**Figure 6:**
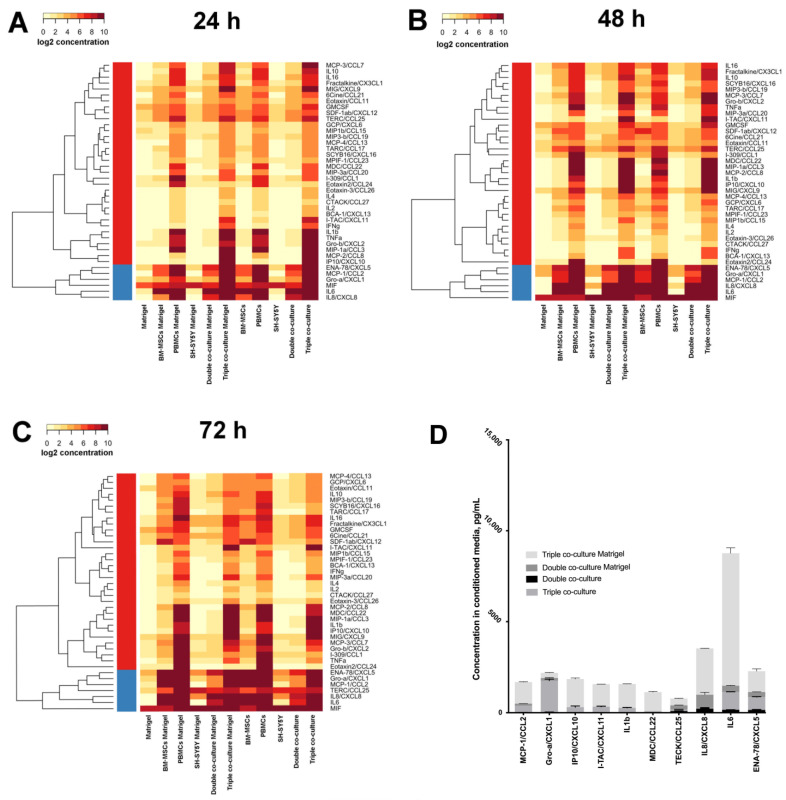
Heat map of cytokine concentrations after 24 (**A**), 48 (**B**), and 72 (**C**) hours of incubation. Data are presented as the logarithm (log_2_) of the mean value in the experiment. The overall comparison of the cytokines with the highest concentration for double and triple co-cultures (**D**).

To prepare the cytokine assay, unstained BM-MSCs and SH-SY5Y cells (double co-culture) or BM-MSCs, PBMCs, and SH-SY5Y cells (triple co-culture) were co-cultivated on plastic or Matrigel for 24, 48, and 72 h. Next, the CM was collected for multiplex analysis. It was shown that Matrigel is also a source of a number of cytokines/chemokines: ENA-78/CXCL5, eotaxin/CCL11, eotaxin-2/CCL24, fractalkine/CX3CL1, GCP/CXCL6, GM-CSF, Gro-α/CXCL1, I-309/CCL1, MIF, MIG/CXCL9, MIP-1β/CCL15, SDF-1α+β/CXCL12, and TECK/CCL25. The highest concentration was observed for the MIF cytokine (222 ± 20.6 pg/mL). Moreover, the BM-MSCs demonstrated a high baseline value of the IL6 cytokine in each experimental group (Figure 6).

The highest concentration (≥200 pg/mL) and the deepest red color blocks on the heatmap (Figure 6) of cytokines in CM of the monocultured BM-MSCs were found for ENA-78/CXCL5, GRO-α/CXCL1, MCP-1/CCL2, IL6, and IL8/CXCL8, on plastic and on Matrigel. In the CM of the double co-culture, the highest concentrations of the following cytokines were observed: ENA-78/CXCL5, TECK/CCL25, IL6, and IL8/CXCL8, on plastic and on Matrigel. The concentrations of IL8/CXCL8 and IL6 were higher on Matrigel than on plastic. In the CM collected from the triple co-culture on plastic and Matrigel, the highest secretion of the following cytokines was observed: MCP-1/CCL2, MIP-1α/CCL3, MCP-2/CCL8, MDC/CCL22, IL1β, IL6, GRO-α/CXCL1, ENA-78/CXCL5, IL8/CXCL8, MIG/CXCL9, IP10/CXCL10, and I-TAC/CXCL11.

### 3.3. Co-Culture of BM-MSCs, SH-SY5Y Cells, and PBMCs Is more Resistant to the Cytotoxic Effect of Cisplatin Than the Co-Culture of BM-MSCs and SH-SY5Y Cells

**Figure 7:**
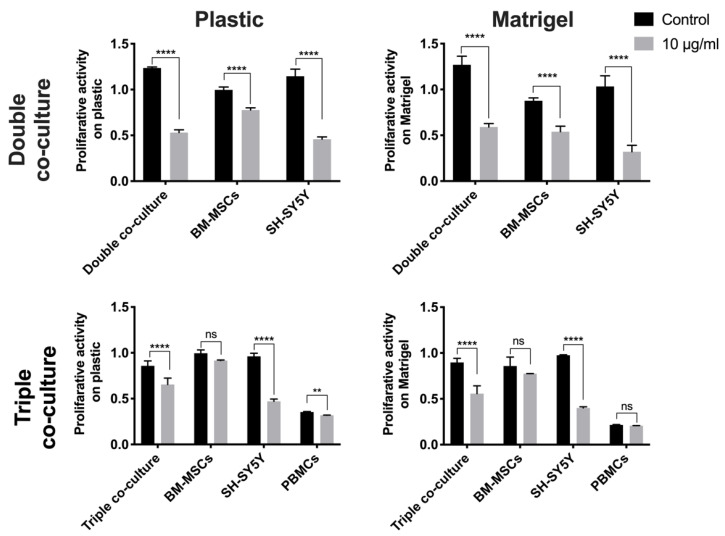
Analysis of the proliferation activity of BM-MSCs and SH-SY5Y cells after incubation with CDDP (10 µg/mL) for 72 h. The results are presented as the mean ± SD (n = 5); **—*p* < 0.01; ****—*p* < 0.0001; ns—no statistically significant difference.

Cisplatin (CDDP) is an antitumor drug containing the heavy metal platinum. CDDP possesses properties similar to that of bifunctional alkylating agents, forming interstitial crosslinks in DNA, thereby interfering with its functions, which leads to cell death [15]. To evaluate the cytotoxic effect of CDDP on co-cultured BM-MSCs and SH-SY5Y cells, the viability of the individual cell populations and co-culture was determined after 72 h of incubation with CDDP 10 μg/mL by MTS assay (Figure 7).

The proliferative activity of the cells in the double co-culture on plastic was decreased by 56.5% relative to co-cultured cells not treated with CDDP. The proliferation rate of SH-SY5Y cells cultivated as a monoculture on plastic and on Matrigel in the presence of CDDP was decreased by 60.0% and 57.8%, respectively, relative to the proliferative activity of native SH-SY5Y cells. The proliferative activity of the double co-culture was decreased by 52.8% (on Matrigel) and 56.5% (on plastic) relative to the proliferative activity of the co-culture not treated with CDDP. The proliferative activity of cells in the triple co-culture after incubation with CDDP was decreased by 23.8% (on plastic) and by 37.8% (on Matrigel) relative to the proliferative activity of the triple co-culture of cells not treated with cisplatin (Figure 7).

### 3.4. Cisplatin Treatment and Co-Cultivation Change Levels of BAX, BCL2, BCL2L1, CAV1, CASP3, and RAC1 Gene Expression in SH-SY5Y

**Figure 8:**
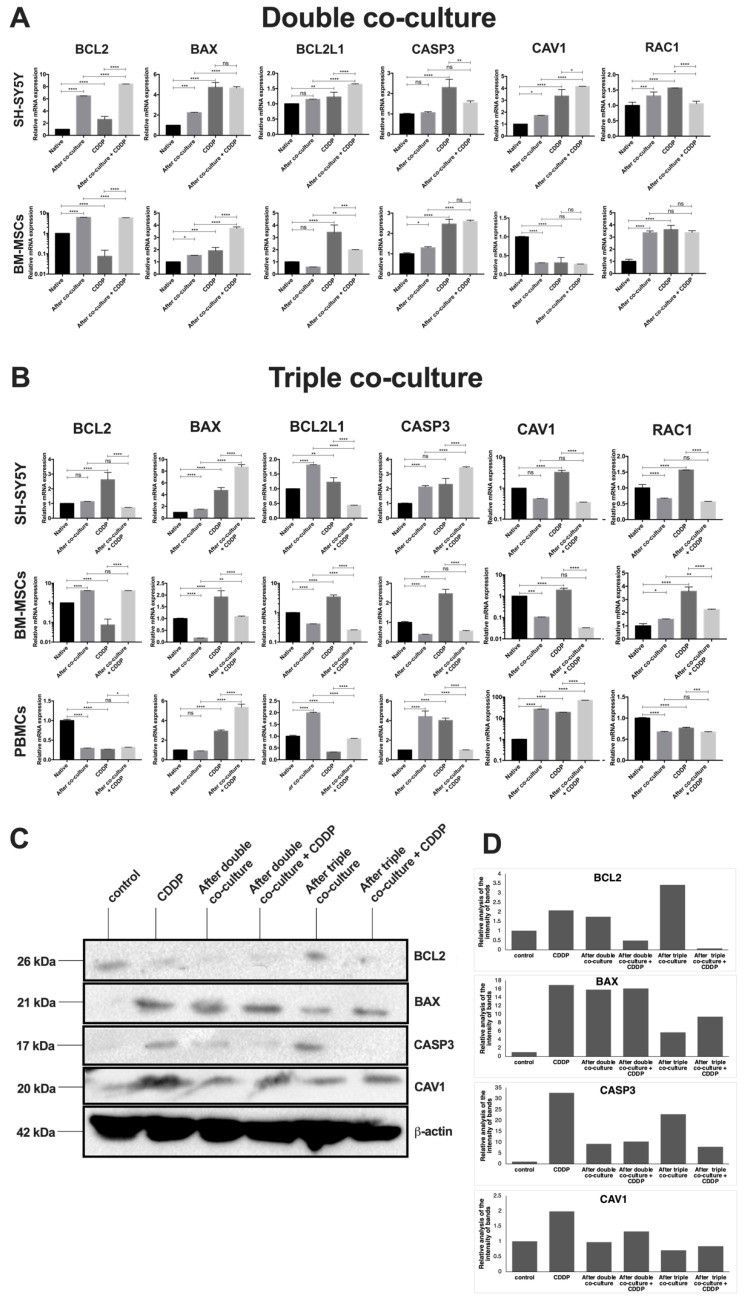
Analysis of the relative mRNA expression of *BCL2, BAX, BCL2L1, CASP3, CAV1,* and *RAC1* genes in BM-MSCs, SH-SY5Y cells, and PBMCs after 72 h of incubation with CDDP at a concentration of 10 μg/mL via double (**A**) and triple (**B**) co-cultures. Western blot analysis for BCL2, BAX, CASP3, and CAV1 proteins in SH-SY5Y cells after co-cultivation and CDDP treatment (**C**). The relative analysis of the intensity of protein bands (**D**). Control—CDDP untreated cells. The data were obtained using qPCR. * *p* < 0.05, ** *p* < 0.01, *** *p* < 0.001, **** *p* < 0.0001, ns—no statistically significant difference.

We investigated the effect of CDDP in the co-cultures of BM-MSCs and SH-SY5Y as well as of BM-MSCs, PBMCs, and SH-SY5Y on *BAX, BCL2, BCL2L1, CAV1, CASP3,* and *RAC1* gene mRNA expression. In order to prevent the mixing of fluorescence spectra and to be able to obtain a larger number of cells for qPCR analysis in the future, we used SH-SY5Y cells transduced with the lentivirus encoding *gfp*. Co-cultures were established by mixing cells in a 1:1 ratio. After 72 h of incubation, cisplatin was added at 10 μg/mL. This concentration was chosen because of the most pronounced cell response in the experiment described above. After an additional 72 h of incubation, FACS sorting was performed using the fluorescent labeling of SH-SY5Y and the differences in the sizes between PBMCs and BM-MSCs in the triple co-culture. The expression levels of *BAX, BCL2, BCL2L1, CAV1, CASP3,* and *RAC1* gene mRNA were determined by qPCR. The relative expression levels of target genes were calculated using 18S rRNA as a reference gene. The expression levels of the proteins BAX, BCL2, CAV1, and CASP3 in SH-SY5Y cells were examined using Western blot analysis (Figure 8).

The mRNA levels of *BCL2, BCL2L1, BAX, CASP3, CAV1*, and *RAC1* genes were changed in SH-SY5Y cells after incubation in monoculture with CDDP, after co-cultivation with BM-MSCs, and after co-cultivation in the presence of CDDP. The levels of BAX and CAV1 proteins after co-cultivation with BM-MSCs and after the co-cultivation in the presence of CDDP were examined using Western blot. The mRNA levels of the *BAX* and the *CASP3* genes were increased in SH-SY5Y cells after incubation with CDDP, after co-cultivation with BM-MSCs and PBMCs, and after the incubation of a triple co-culture with CDDP. The mRNA level of the *BCL2* gene was increased after co-cultivation but decreased after the incubation of the triple co-culture with CDDP. The mRNA levels of the *BCL2L1, CAV1*, and *RAC1* genes were increased after the incubation of SH-SY5Y cells with CDDP but decreased after the co-cultivation and incubation of a triple co-culture with CDDP. The levels of BAX and CAV1 proteins in all experimental groups of SH-SY5Y cells, as well as BCL2 and CASP3 proteins in SH-SY5Y cells after the co-cultivation with BM-MSCs and PBMCs, were determined using Western blot analysis.

We also examined changes in the mRNA levels in BM-MSCs and PBMCs. The transcription of the *BAX* and *RAC1* genes was increased in BM-MSCs after double co-culturing and double co-culturing in the presence of CDDP. The mRNA levels of the *BCL2* and *RAC1* genes were reduced in PBMCs after co-cultivation with BM-MSCs and SH-SY5Y cells, after the incubation of PBMCs with CDDP, and after the co-cultivation in the presence of CDDP, and the level of the *CAV1* gene mRNA was increased in these experimental groups. The mRNA level of the *BAX* gene was decreased after the incubation of PBMCs with CDDP but increased in the remaining experimental groups. The mRNA level of the *BCL2L1* gene was increased in PBMCs after incubation with CDDP but decreased after co-culture and CDDP treatment. The mRNA level of the *CASP3* gene was decreased after co-cultivation with CDDP and increased in the other groups.

## 4. Discussion

We have analyzed the interaction of BM-MSCs, SH-SY5Y cells, and PBMCs in co-cultures. It was found that BM-MSCs and SH-SY5Y tumor cells in the double co-culture, as well as BM-MSCs, SH-SY5Y cells, and PBMCs in the triple co-culture, self-organize in the co-culture, interacting by cell-to-cell contact, and also with the help of vesicular transport and exchange membrane and cytoplasmic components. As a result of the cell self-organization, various structures were formed: saucer-like structures on plastic and spheroid-like structures on Matrigel in vitro, which included mixed cultured cell types.

Previous co-culture studies of human MSCs derived from third molar tooth germs (hTGSCs) and SH-SY5Y cells showed saucer-like formations after 72–96 h of co-cultivation; hTGSCs were located in the center and surrounded by SH-SY5Y cells [16]. Thus, the spheroid formations described in the present study were not previously observed, suggesting that the type of self-organization of cells in a co-culture can be determined by the source of MSCs and/or the individual characteristics of the stem cell donor. Further, studies to characterize the effects of different sources of stem cells are required. However, the formation of different self-organization patterns potentially reflects the nature of the interaction between the tumor and stromal cells during tumor metastasis in the body. In this way, studies of cell self-organization in models that simulate the three-dimensional (3D) structures of tumor tissues can increase the effectiveness of the screening of antitumor drugs.

At present, the NCI60 test system is most often used for primary antitumor drug screening, which includes testing the substances on a panel of 60 human tumor cell lines [17]. Synthetic compounds or natural substances were selected for their ability to suppress growth or cause cell death in in vitro cultures. However, this test does not take into account the contribution of TME factors, intercellular communication autocrine and paracrine regulation and tumor interaction, or immune and stromal cells with an ECM, all of which have a significant effect on the progression and properties of the tumor in the body [18].

Currently, 3D models of cell culture differ from conventional monolayer cultures by growing cells either in a 3D environment or within a matrix or framework with 3D architecture. Within three dimensions, cells from the multilayer structures, as demonstrated in our study, form a recapitulated model of the tumor tissue. These more physiologically relevant 3D models obtained in vitro can potentially be used to test the activity of antitumor drugs. It was shown that studies used the same spheroid modeling technique to test anticancer drug delivery by liposomes with promising results. Treatment with 5-fluorouracil, oxaliplatin, and folinic acid at IC50 values corresponding to those used in the 2D culture system decreased the viability of the HT-29 human colon adenocarcinoma cells in the 3D culture systems by 50% following their encapsulation into liposomes [19]. Thus, 3D and spheroidal models based on the use of two or more cell types, as well as an ECM analog, have a great potential for creating test systems for screening the potential of antitumor drugs since they reproduce the microenvironment and the architecture of the tumor tissue.

The exchange between membrane components indicates an intensive cell-to-cell interaction and communication of BM-MSCs and SH-SY5Y cells by vesicular transport. An investigation conducted using prostate cancer cells showed the high vesicle transfer between the stromal and cancer cells in co-culture [20]. This phenomenon plays a dual role in cancer progression, leading to the emergence of drug resistance and the enhancement of tumor aggression. In addition, however, the high vesicle transfer between the stromal and cancer cells can open new therapeutic approaches for targeted drug delivery to tumors [21]. It is known that MVs produced by tumor cells carry different proteins, regulatory microRNAs, and functional mRNAs for interaction with stromal microenvironment cells and the modulation of their functions [22]. MSC-derived MVs also modulate the functions of tumor cells. For example, MSCs can induce CD90 expression in breast cancer cells MDA-MB-231 [23], resulting in a more complex and heterogeneous tumor [24]. In addition, fluorescence in two spectra indicates the formation of hybrid cells by the spontaneous fusion of MSCs and tumor cells. This phenomenon has already been described in the literature. For example, Mendel et al. showed the formation of hybrid cells after the spontaneous fusion of MSCs and breast cancer cells MDA-MB-231 in co-culture in vitro [25]. In our previous study, we also found that after the co-culture of hTGSCs with SH-SY5Y cells labeled with fluorescent vital dyes, some cells had a dual fluorescence spectrum [16]. In the investigation of co-culturing MSCs, peripheral blood mononuclear cells and adenocarcinoma cells (HeLa) showed a high degree of cell interaction through the exchange of membrane components, which were expressed as the co-localization of fluorescence spectra [14]. It may be concluded that these processes are systematic for co-cultured cells.

Such a result may be explained by the fact that MSCs play a supporting role in tumorigenesis through the secretion of factors such as IL6, IL8, VEGF, platelet-derived growth factor (PDGF), fibroblast growth factor (FGF)-7, TGF-β, and vimentin, supporting the growth and chemoresistance of the tumor [26]. The role of MSCs in supporting the growth of tumor cells has been observed in other studies. For example, under conditions of oxidative stress induced by H_2_O_2_ treatment, the viability of SH-SY5Y cells was increased by 2.1–3.5 times in the presence of MSCs [16].

MSCs are known to be resistant to the cytotoxic effect of chemotherapeutic drugs, such as CDDP [27,28], with a concentration of 5 μg/mL causing no significant ultrastructural changes in MSCs [29]. Given the major impact of acquired resistance to CDDP in patients [30], strategies to target MSCs, particularly their ability to support tumor cell growth, prove to be effective in reducing the formation of tumor resistance to chemotherapy. It has been shown that CDDP influences the secretory phenotype and behavior of MSCs in vitro. For example, the pretreatment of MSCs with CDDP resulted in changes in the phosphorylation profiles of many kinases, as well as in the enhancement of the secretion of IL6 and IL8 cytokines. These changes in the cytokine and kinase phosphorylation profile of MSCs resulted in an increase in breast cancer cell chemoresistance [31]. The higher proliferative activity of the triple co-culture in comparison with the double co-culture can be explained by the secretion of chemokines by immune cells, which contribute to the preservation of the higher proliferative activity of the cells in the triple co-cultures. For example, TERC/CCL25 is associated with an increase in the proliferative potential of tumor cells [32,33]. It is possible that such factors represent potential targets to reduce chemoresistance mediated by MSCs.

The increase in chemokine secretion after 48 and 72 h of co-cultivation signals the development of cell–cell interactions and the exhibition of the pro-oncogenic activity of MSCs in co-culture with SH-SY5Y cells. It was shown that a decrease in the expression level of CXCL12 in MSCs contributed to the metastasis of tumor cells [34]. Additionally, an elevated concentration of IL6 in serum was associated with a negative prognosis of the tumor in patients with different cancer types [35]. Fang et al. described a negative correlation between the expression level of CXCL16 in tumor cells and between invasiveness and cells, specifically migration, the suppression of CXCL16 secretion in MCF-7 cells, and increased cell invasiveness [36].

We showed that PBMCs are the source of a large number of cytokines; in addition, similar cytokines (I-TAC/CXCL11, MCP-2/CCL8, MDC/CCL22, MIG/CXCL9, MIP-1α/CCL3, IL1β, IP-10/CXCL10, ENA-78/CXCL5, Gro-α/CXCL3, MCP-1/CCL2, IL8/CXCL8, and IL6) were found in high concentrations in the CM collected from the triple co-culture. According to the literature, CXCL11 (also called I-TAC, a ligand for the CXCR3 and CXCR7 receptors) attracted CD8^+^ T cells and NK cells to tumors or other sites of inflammation. It has potential in vivo anti-tumor activity that involves the recruitment of CD8^+^ T cells [37]. CXCL11 fused to the Fc region can act as a potent adjuvant to enhance antigen-specific CD8+ T-cell responses [38]. CXCL11 can also bind to the CXCR3 receptor and mediate endothelial cell inhibition and hence tumor angiogenesis [39]. According to the results of this study, and consistent with previous research, high levels of CXCL8/IL8 and IL6 are secreted primarily by cells of the TME [40] and are associated with a poor survival prognosis for patients with osteosarcoma [41]. In addition, high levels of CXCL8/IL8, IL6, and MCP-1/CCL2 secretion promoted the migration of tumor cells and macrophage-like cells, exacerbating tumor progression [42]. It was also shown that CXCL8/IL8 and IL6 induced the resistance of tumor cells to chemotherapy [43]. Previous research found that the tumor-associated MSCs produced chemokines Gro-α/CXCL1, Gro-β/CXCL2, and IL8, which bound to the CXCR1/2 receptor on the surface of tumor cells and led to the formation of chemoresistance and induced the polarization of macrophages [44]. MSCs secreted IL6, CCL5m, and IP-10/CXCL10 and exhibited increased motility in response to multiple soluble factors produced by macrophages, including IL8, CCL2, and CCL5 [45].

According to the literature, the increased secretion of ENA-78/CXCL5 in tumor tissues is associated with lymphatic metastases and tumor differentiation, with the induction of EMT, as exemplified by gastric cancer cells [46]. It was demonstrated that MCP-3/CCL7 belongs to the group of chemokines involved in inflammatory processes, recruiting macrophages, and triggering cellular senescence [47,48]. The CXCL12 chemokine mediated pro-angiogenic and prometastatic effects through receptors CXCR4 and CXCR7, while the neutralization of this chemokine reduced the angiogenic potential of the tumor [49]. Moreover, the I-309/CCL1 was highly expressed in human breast cancer; in addition, the high expression of this chemokine correlated with the infiltration of immunosuppressive FoxP3^+^ Tregs, which negatively affect patient survival [50]. The secretion of CCL1 by CD11b^+^ CD14^+^ myeloid cells was involved in the infiltration of Tregs [51]. Thus, the high levels of these cytokines in the conditioned medium collected from the triple co-culture demonstrated the pro-tumor activity of a number of cells in the mononuclear fraction.

CXCL8/IL8, ENA-78/CXCL5, IL6, and TERC/CCL25, both in double co-culture and triple co-culture, indicated the formation of a microenvironment with chronic inflammation characteristic of the tumor stroma and the active participation of MSCs in these processes. High concentrations of the inflammatory chemokines in the triple co-culture (I-TAC/CXCL11, IP-10/CXCL10, MCP-1/CCL2, MCP-2/CCL8, MDC/CCL22, MIG/CXCL9, MIP-1α/CCL3, IL1β, ENA-78/CXCL5, Gro-α/CXCL3, IL6, and IL8/CXCL8), predominantly secreted by PBMCs, may indicate the involvement of various cell populations in the formation of chronic inflammation typical of a natural tumor and the antitumor effects of some populations of immune cells from PBMC fractions.

Our results indicated the changes in the mRNA levels of the *BCL2, BCL2L1, BAX, CASP3, CAV1*, and *RAC1* genes as well as levels of the BCL2, BAX, CASP3, and CAV1 proteins associated with apoptosis and the malignant transformation of cells in the co-cultures of tumor, immune, and stromal cells in the presence of CDDP.

BCL2L1 and the BCL2 protein are anti-apoptotic, key regulators of apoptosis, and are actively involved in the regulation of other vital cellular functions. BCL2 is one of the most crucial regulators of apoptosis and autophagy [52]. The overexpression of BCL2 was previously related to the malignant progression of tumors [53]. BCL2 overexpression reduced the CDDP-induced growth inhibition and apoptosis in SKOV3 human ovarian cancer cells [54]. Moreover, some evidence suggests that high levels of BCL2L1 protein expression prevent the death of cancer cells such as glioma [55]. An increase in the mRNA levels of the *BCL2* and *BCL2L1* genes and the detection of protein expression in SH-SY5Y cells may be associated with the antiapoptotic response and the development of drug resistance.

CASP3 is activated during anticancer therapy, leading to cell death [56]. The involvement of CASP3 in the transcriptional regulation of the VEGFR pathway led to an increase in the angiogenesis of the tumor [57]. Thus, an increase in the relative level of CASP3 in SH-SY5Y cells in a triple co-culture can signal both the initiation of apoptosis processes and the development of chemoresistance, leading to an increased metastatic potential of tumor cells. Proapoptotic BAX protein dysregulation also correlates with the progression of cancer and the development of chemoresistance on squamous cell carcinoma of the oral cavity [58]. With an increase in BAX and a decrease in BCL2, the apoptosis of cells of the VX2 liver cancer model was observed [59]. The ratio of BAX and BCL2 expression becomes an important marker of survival in patients with neuroendocrine lung tumors [60]. In the present study, an increase in BAX expression was observed in all experimental groups. However, the BAX protein expression in SH-SY5Y cells after the triple co-culture was higher than in the tumor cells after the double co-culture, which indicates a cytotoxic influence of a number of PBMCs on tumor cells in the triple co-culture.

CAV1 plays a key role in negative feedback to stabilize the MSC phenotype during differentiation. Investigations conducted on murine melanoma models showed strong tumor growth and metastasis formation after undifferentiated MSC transplantation [60]. Thus, the significant decrease in CAV1 expression level in BM-MSCs after co-cultivation with SH-SY5Y cells demonstrated a decrease in the differentiation capacity of BM-MSCs, as evidenced by the recruitment of BM-MSCs to tumor cells. Moreover, the decrease in CAV1 expression may enhance the oncogenic transformation of normal cells, perhaps by disrupting contact inhibition in transformed cells [60]. Recent research has shown that after short-time anticancer drug treatment, the CAV1 expression was increased in cancer cells, which resulted in the enhanced migration and invasion of cancer cells in vivo [61]. Thus, the increase in CAV1 expression in SH-SY5Y after CDDP treatment in the co-culture and monoculture confirmed the magnification of the CAV1-dependent invasive, migratory, and metastatic properties of the cancer cells.

RAC1 is a member of the small GTPases involved in signaling pathways regulating the actin cytoskeleton, cell cycle, and other cellular processes [62]. A significant correlation between high RAC1 activity in clinical specimens and the worse survival of patients after surgery was associated with high metastasis and tumor chemoresistance [63]. In addition, an increase in RAC1 expression mediates the phenomenon of cell fusion [64], which leads to increased cell heterogeneity in tumors, resulting in the increased metastatic behavior of the cells [65]. The increase in RAC1 expression indicates the diverse interactions between different cells, which exacerbate the degree of tumor malignancy. Our findings indicate the active participation of PBMCs in the growth inhibition of tumor cells, reducing their metastatic capacity and suppressing the tumor-supporting properties of BM-MSCs.

## 5. Conclusions

The microenvironment plays a key role in tumor progression, metastasis, and the development of therapeutic resistance. In the present work, the mechanisms of key cell interactions involved in the tumor process were studied. Findings indicated that BM-MSCs, SH-SY5Y cells, and PBMCs interacted in co-cultures using vesicular transport and exchanging membrane/cytoplasmic components. In addition, the co-culture of BM-MSCs, SH-SY5Y cells, and PBMCs have a higher resistance to the cytotoxic effect of CDDP, in contrast to the culture containing only BM-MSCs and SH-SY5Y cells, as well as the monocultures of SH-SY5Y cells. In the process of the co-cultivation of the cells in double and triple co-cultures, differences in the composition of cytokines were observed, mainly due to the different compositions of the co-cultures and the response of the cells to the long-term co-cultivation. After the co-cultivation of BM-MSCs, SH-SY5Y cells, and PBMCs, the levels of the mRNA of genes involved in apoptotic processes as well as the levels of the mRNA of genes associated with differentiation and migration changed. The overexpression of BCL2 and CASP3 proteins in SH-SY5Y cells after the triple co-cultivation may be a sign of the initiation of anti-apoptotic compensatory processes in response to the cytotoxic effect of some PBMC populations. Despite a possible decrease in the antiapoptotic resistance of SH-SY5Y cells, the proliferative activity of the cells in the triple co-culture was higher than in the double ones and the monocultures of neuroblastoma cells, which could be explained by an increased concentration of a number of cytokines (CXCL8/IL-8, I-TAC/CXCL11, IP10/CXCL10, MDC/CCL22, IL1β, ENA-78/CXCL5, Gro-a/CXCL1, MCP-1/CCL2, TECK/CCL25, and IL-6) secreted by PBMCs and BM-MSCs. These secretions can induce a high level of proliferation despite the cytotoxic effects of CDDP. The 3D structures imitating the microenvironment of tumor tissue can be used as test systems capable of improving the quality of anticancer screening.

## Figures and Tables

**Table 1:** Primer and probe sequences of related genes for qPCR.

Target Gene	Forward Primer (5′−3′)	Reverse Primer (5′−3′)	TaqMan Probe (5′−3′)
18S rRNA	GCCGCTAGAGGTGAAATTCTTG	CATTCTTGGCAAATGCTTTCG	[HEX] ACCGGCGCAAGACGGACCAG [BH2]
BCL2	GTGGATGACTGAGTACCTGAAC	GCCAGGAGAAATCAAACAGAGG	[FAM] CAGGATAACGGAGGCTGGGATGC [BH1]
BCL2L1	GACATCCCAGCTCCACATC	GTTCCCATAGAGTTCCACAAAAG	[FAM] CCCCAGGGACAGCATATCAGAGC [BH1]
RAC1	GGTGAATCTGGGCTTATGGG	TCAGGATACCACTTTGCACG	[FAM] TTTGCTTTTCCCTTGTGAGTCCTGC [BH1]
CAV1	CCTTCCTCAGTTCCCTTAAAGC	TGTAGATGTTGCCCTGTTCC	[FAM] TCCTCACAGTTTTCATCCAGCCACG [BH1]
CASP3	CCTACAGCCCATTTCTCCATAC	GCTTCACTTTCTTACTTGGCG	[FAM] CCCTGGCAGCATCATCCACACATA [BH1]
BAX	GACATGTTTTCTGACGGCAAC	AAGTCCAATGTCCAGCCC	[FAM] CTGGCAAAGTAGAAAAGGGCGACAAC [BH1]

## Data Availability

Not applicable.
